# Post-Operative Complications and Nipple Necrosis Rates Between Conventional and Robotic Nipple-Sparing Mastectomy

**DOI:** 10.3389/fonc.2020.594388

**Published:** 2021-01-08

**Authors:** Jeea Lee, Hyung Seok Park, Haemin Lee, Dong Won Lee, Seung Yong Song, Dae Hyun Lew, Jee Ye Kim, Seho Park, Seung Il Kim

**Affiliations:** ^1^ Department of Surgery, Yonsei University College of Medicine, Seoul, South Korea; ^2^ Department of Plastic and Reconstructive Surgery, Yonsei University College of Medicine, Seoul, South Korea

**Keywords:** breast neoplasms, robotic mastectomy, nipple-sparing mastectomy, minimal invasive surgery, nipple necrosis

## Abstract

**Purpose:**

This study is to directly compare surgical outcomes between conventional nipple-sparing mastectomy (CNSM) and robot-assisted nipple-sparing mastectomy (RNSM).

**Materials and Method:**

For this case–control study, 369 cases of 333 patients who underwent CNSM or RNSM with immediate reconstruction between November 2016 and January 2019 at Severance Hospital in Seoul, Republic of Korea were reviewed. Patients with stage IV breast cancer (n = 1), receiving neoadjuvant chemotherapy (n = 43), or subjected to previous operations (n = 14) or radiotherapy on the breasts were excluded. The main outcomes were comparing rates of post-operative complications, of high-grade post-operative complications as defined by the Clavien-Dindo classification, and nipple necrosis between the CNSM and the RNSM groups.

**Results:**

A total of 311 cases, including 270 CNSMs and 41 RNSMs, were analyzed. The rates of post-operative nipple necrosis (*p* = 0.026, 2.4 *vs.* 15.2%) and of high-grade post-operative complications (*p* = 0.031, 34.8 *vs.* 17.1%) in the RNSM group were significantly lower than those in the CNSM group.

**Conclusion:**

RNSM was associated with lower rates of high-grade post-operative complications and nipple necrosis than CNSM for patients with small breast volumes and less ptotic breasts.

## Introduction

Nipple-sparing mastectomy (NSM) has been widely applied to women with early breast cancer or *BRCA 1/2* mutations (1–4). Because NSM preserves the nipple areolar complex (NAC) and overlying skin, NSM results in better cosmetic outcomes coupled with oncologic safety for those patients, compared to conventional total mastectomy or skin-sparing mastectomy (4–9).

Nipple necrosis is one of the most common complications after NSM (1, 2, 10, 11). Previous studies reported 0–48% of nipple ischemia or nipple necrosis in patients undergoing NSM with immediate reconstruction (1, 12). In order to reduce nipple ischemia or necrosis, various techniques have been proposed in previous studies (12, 13). Rusby et al. showed that placement of incisions far from the NAC and reconstruction using a tissue expander (T/E) reduced the risk of NAC necrosis (12). Petit et al. reported that leaving a layer 5 mm of glandular tissue beneath the NAC for preserving its blood supply is beneficial to reduce NAC necrosis (13). However, there is no universal solution for reducing nipple necrosis after NSM.

Many surgeons have tried to develop various incisions in NSM to deliver better cosmetic outcomes (14–16). Robot-assisted nipple-sparing mastectomy (RNSM) is a procedure that uses robotic systems through axillary or lateral incisions, which results in no scars in the overlying skin. A previous study reported that RNSM presented with low rates of nipple necrosis (17–19). However, there has been, to our knowledge, a lack of comparisons between RNSM and conventional NSM (CNSM) in terms of nipple necrosis rates.

This study aimed to evaluate nipple necrosis rates between RNSM and CNSM. Additionally, grades and rates of complications after the two procedures were analyzed and compared.

## Materials and Methods

### Patients

**Figure 1:**
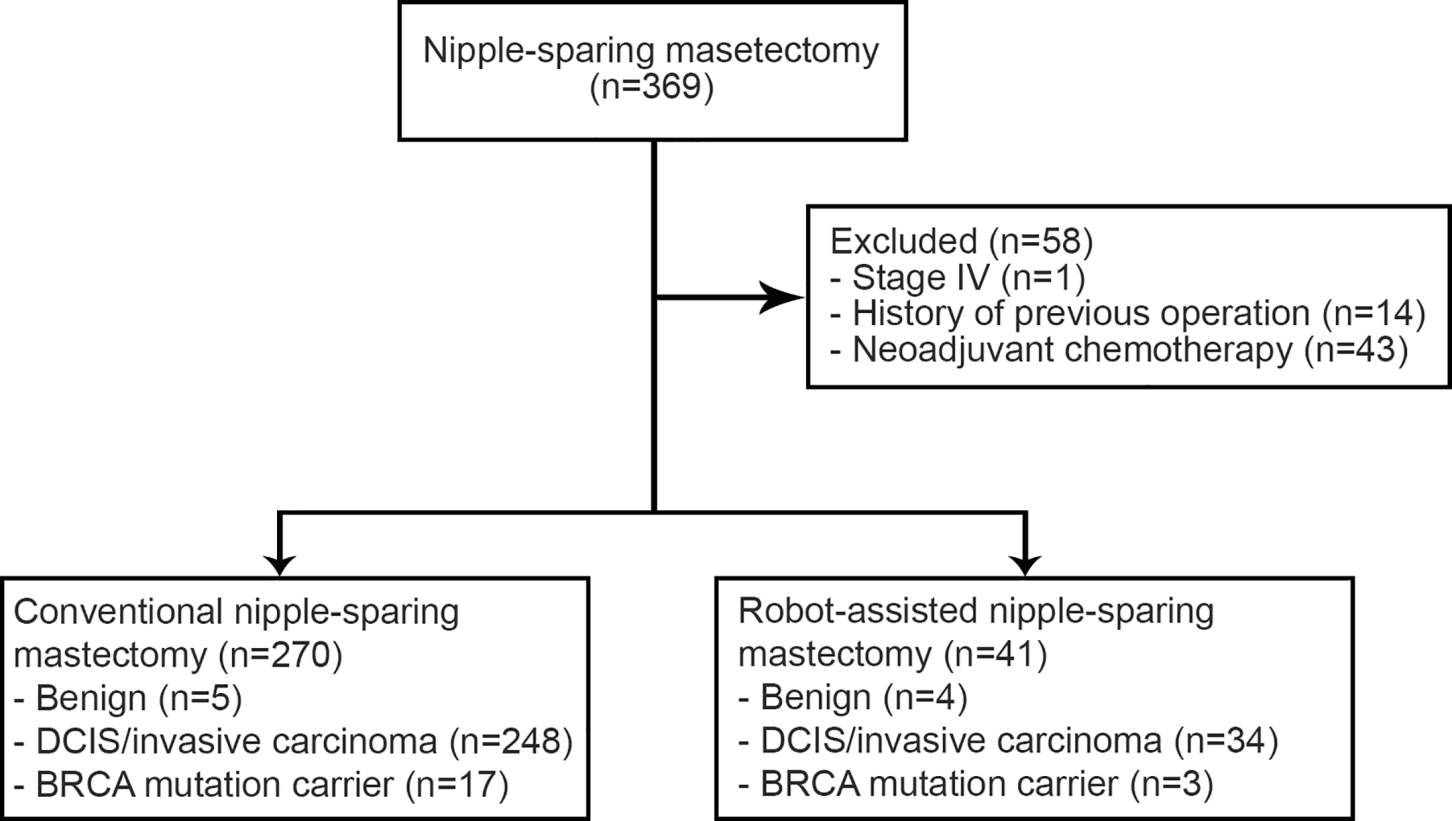
Schematic diagram of the study population.

A total of 333 patients in the present study had undergone CNSM or RNSM between November 2016 and January 2019 at Severance Hospital, Seoul, Korea. Their medical records and post-operative photographs taken by plastic surgeons were retrospectively reviewed. The photographs were taken on 1, 2, 3, 5, 7, and 9 days after the operation of autologous reconstruction routinely. After a prosthetic reconstruction, post-operative photographs were taken on 1, 2, 4, 6, and 8 days after the operation. In an outpatient department, plastic surgeons take the photographs as needed. Exclusion criteria were the presence of stage IV disease (n = 1), treatment with neoadjuvant chemotherapy (n = 43), and previous operation or radiation history (n = 14). This resulted in a total of 311 cases, 270 cases with CNSM and 41 cases with RNSM, from 275 patients being enrolled in the study (**Figure 1**). Among them, 36 patients underwent either bilateral CNSM or RNSM. There was no male patient in this study because patients who underwent immediate reconstruction after mastectomy were collected.

Clinicopathologic features, including age, BMI, breast volume, ptosis, disease entities, TNM stage, estrogen and progesterone receptor, human epidermal growth factor receptor (HER) 2 status, Ki 67 levels, adjuvant therapies, reconstruction methods, duration of hospital stays, and operation times were analyzed. Post-operative complications through 1–28 months, including nipple ischemia or necrosis, skin ischemia or necrosis, infection, bleeding, lymphedema, limitation of shoulder movement, contracture, seroma, wound dehiscence, and arterial thrombus, were also analyzed. Nipple ischemia in this study was defined as a clinical ischemic color change in the NAC. Nipple necrosis was defined as full-thickness necrosis of the NAC requiring surgical intervention (1). Grades of post-operative complications were analyzed according to the Clavien-Dindo classification (20).

### Procedures

**Figure 2:**
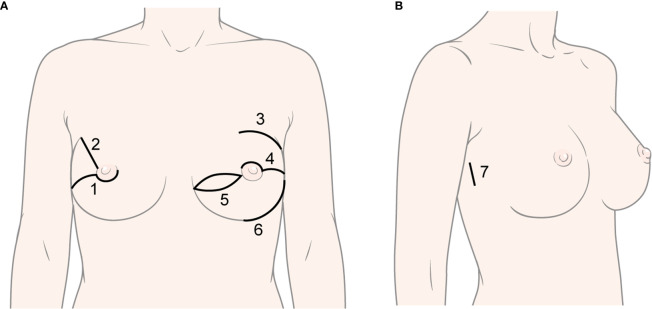
Various types of incisions in conventional nipple-sparing mastectomy and robot-assisted nipple-sparing mastectomy. **(A)** Types of incision in conventional nipple-sparing mastectomy: 1) Lower-periareolar incision with/without extension, 2) Radial incision, 3) Curvilinear incision, 4) Upper-periareolar incision with/without extension, 5) Elliptical incision, 6) Inframammary fold incision; **(B)** Type of incision in robot-assisted nipple-sparing mastectomy: 7) Lateral incision.

CNSM was performed using various methods by three breast surgeons (**Figure 2**). Immediate reconstruction, including tissue expander (T/E), direct-to-implant (DTI), Latissimus dorsi (LD) flap, and transverse rectus abdominis musculocutaneous (TRAM) flap, was performed according to surgeons’ and patients’ preferences by three plastic surgeons. A deep inferior epigastric perforator flap was included in the TRAM flap. RNSM was performed *via* single axillary or lateral incision by a breast surgeon. Gas or gasless technique in robotic mastectomy was applied to patients with early breast cancer or *BRCA* mutations (17–19, 21). The detailed techniques were described in previous studies (17–19, 21). T/E insertion or DTI was applied for immediate reconstruction in those patients (19, 21).

### Pathologic Evaluations

Estrogen receptor (ER), progesterone receptor (PR), HER2 status, and Ki 67 levels were analyzed by immunohistochemistry (IHC), as described in previous studies (22). In brief, positivity for ER and PR was defined as ≥1% nuclear staining in IHC. HER2 2+ in IHC and amplification in fluorescence *in situ* hybridization/silver *in situ* hybridization or 3+ in IHC were considered overexpression according to ASCO/CAP guidelines (23). The cut-off values for Ki 67 staining for low and high proliferative index were < and ≥14% staining in IHC, respectively (24). TNM stage was classified according to anatomic stage as in the AJCC 8^th^ edition. Nipple margins were reviewed from both intra-operative frozen and post-operative permanent pathologic evaluations.

### Adjuvant Therapies

Chemotherapy, endocrine therapy, and radiation therapy were delivered according to standard guidelines or physicians’ preferences (25). Patients with HER2-positive disease and tumor sizes ≥1 cm routinely received adjuvant trastuzumab therapy.

### Statistics

A learning curve of RNSM for total operation time was analyzed using three-day moving average curves (3D-MAC), and the cumulative sum (CUSUM) technique. 3D-MAC is used to analyze the existence of a learning curve (26). This simple moving average is defined as the mean value of previous 3 days data points (27). The CUSUM technique is a statistical method to assess the learning curve quantitatively and to calculate the sequential difference between the individual and the mean value of all data (28). The CUSUM is estimated by $\mathrm{CUSUM}=\sum_{i=1}^{n}\left(xi-\mu \right)$ , where *xi* is an individual operation time, and *µ* is the mean value of overall operation time (29).

Categorical variables were analyzed using either Chi-square test or Fisher’s exact test, if indicated. Continuous variables were analyzed using either Student’s t test or Mann–Whitney test, if indicated. All tests were two-sided. Multivariate analysis was performed using binary regression with backward elimination (conditional) to evaluate risk factors related with high-grade complications (Clavien-Dindo classification ≥grade III). A *p-*value less than 0.05 was considered to be statistically significant. All statistical analyses were performed using the SPSS software, version 25 (SPSS Inc., Chicago, IL). We did not use a statistical matching technique due to the limited sample size. Missing values were imputed as null values.

### Ethics

This study was approved by the institutional review board at Severance Hospital (4–2019–0510).

## Results

The clinicopathologic features of the enrolled patients are shown in **Table 1**. The mean age of patients was 45.93 ± 8.34 (data not shown). There were no differences in clinicopathologic features between the CNSM and RNSM groups, except in breast volumes, laterality, and ptosis. Ptotic breasts were more frequent and breast volumes were larger in the CNSM group. Others subgroup in *BRCA* mutation included three cases with *PALB2* mutations (**Table 1**).

**Table 1:** Clinicopathologic characteristics of the study population.

		CNSM	RNSM	*p*-value^b^
		(n = 270)	(n = 41)	
Age (years)		46 ± 8.0	44 ± 10.0	0.075**^c^**
BMI (kg/m^2^)		22.5 ± 3.1	21.7 ± 2.3	0.065**^c^**
Breast volume (g)		428 ± 222.0	326 ± 143.0	0.002**^c^**
Laterality	Unilateral	216 (80.0)	23 (56.1)	0.001
	Bilateral	54 (20)	18 (43.9)	
Ptosis	Normal	136 (50.4)	32 (78.0)	0.004
	Mild	56 (20.7)	8 (19.5)	
	Moderate	36 (13.3)	0 (0.0)	
	Severe	38 (14.1)	1 (2.4)	
	Pseudoptosis	2 (0.7)	0 (0.0)	
*BRCA1* mutation	No	89 (81.7)	16 (94.1)	0.913
	Yes	11 (10.1)	1 (5.9)	
	VOUS	6 (5.5)	0 (0.0)	
	Others	3 (2.8)	0 (0.0)	
*BRCA2* mutation	No	89 (81.7)	10 (58.8)	0.050
	Yes	12 (11.0)	6 (35.5)	
	VOUS	5 (4.6)	1 (5.9)	
	Others	3 (2.8)	0 (0.0)	
Diagnosis	Benign	5 (1.9)	4 (9.8)	0.069
	DCIS	63 (23.3)	9 (22.0)	
	Invasive carcinoma	185 (68.5)	25 (61.0)	
	*BRCA* mutation carrier	17 (6.3)	3 (7.3)	
ER^a^	Negative	49 (19.8)	3 (8.8)	0.123
	Positive	199 (80.2)	31 (91.2)	
PR^a^	Negative	64 (25.8)	8 (23.5)	0.775
	Positive	184 (74.2)	26 (76.5)	
HER2^a^	Negative	174 (76.3)	21 (63.6)	0.117
	Positive	54 (23.7)	12 (36.4)	
Ki 67^a^	Low (<14%)	108 (44.3)	13 (38.2)	0.632
	High (≥14%)	136 (55.7)	21 (61.8)	
Histologic grade^a^	Grade I	59 (23.8)	5 (14.7)	0.445
	Grade II	144 (58.1)	21 (61.8)	
	Grade III	45 (18.1)	8 (23.5)	
T^a^	Tis	67 (27.0)	11 (32.4)	0.615
	T1	144 (58.1)	20 (58.8)	
	T2	37 (14.9)	3 (8.8)	
N^a^	N0	210 (86.1)	30 (88.2)	0.653
	N1	29 (11.9)	3 (8.8)	
	N2	4 (1.6)	1 (2.9)	
	N3	1 (0.4)	0 (0.0)	
TNM stage^a^	0	68 (27.4)	8 (23.5)	0.766
	I	126 (50.8)	20 (58.8)	
	II	48 (19.4)	5 (14.7)	
	III	6 (2.4)	1 (2.9)	
Adjuvant chemotherapy^a^	No	167 (67.3)	23 (67.6)	0.971
	Yes	81 (32.7)	11 (32.4)	
Radiotherapy^a^	No	220 (88.7)	30 (88.2)	0.935
	Yes	28 (11.3)	4 (11.8)	
Hormone therapy^a^	No	58 (23.4)	5 (14.7)	0.254
	Yes	190 (76.6)	29 (85.3)	
Target therapy^a^	No	231 (93.1)	29 (85.3)	0.161
	Yes	17 (6.9)	5 (14.7)	
Recurrence^a^	No	246 (99.2)	41 (100.0)	> 0.999
	Yes	2 (0.8)	0 (0.0)	

Values are represented as mean ± SD or number (percentage).

BMI, body mass index; CNSM, conventional nipple-sparing mastectomy; DCIS, ductal carcinoma in situ, ER, estrogen receptor; HER, human epidermal growth factor receptor; PR, progesterone receptor; RNSM, robot-assisted nipple-sparing mastectomy; VOUS, variants of unknown significance.

^a^29 cases of benign disease or BRCA mutation carriers were not included (n = 282).

^b^Chi-square test or Fisher’s exact test.

^c^Student’s t test or Mann–Whitney test.

Post-operative outcomes, including length of hospital stay and operation times are shown in **Table 2**. The length of hospital stay in the RNSM group was greater than in the CNSM group (*p* < 0.001, 14 ± 4 *vs.* 12 ± 3 days), and the same held for total operation time (*p* < 0.001, 308.9 ± 75.5 *vs.* 303.9 ± 195.9 min). Mastectomy time was longer in the RNSM group than the CNSM group (*p* < 0.001, 181.5 ± 44.7 *vs.* 104.5 ± 40.5 min), Reconstruction time was longer in the CNSM group than the RNSM group (*p* = 0.019, 196.8 ± 182.5 *vs.* 140.5 ± 52.5 min).

**Table 2:** Surgical methods and post-operative outcomes.

		CNSM	RNSM	*p*-value^b^
		(n = 270)	(n = 41)	
Hospital stay (days)		12 ± 3	14 ± 4	0.001^c^
Total operation time (min)		303.9 ± 195.9	308.9 ± 75.5	< 0.001^c^
Mastectomy time (min)		104.5 ± 40.5	181.5 ± 44.7	< 0.001^c^
Console time (min)		–	64 ± 40	–
Reconstruction time (min)		196.8 ± 182.5	140.5 ± 52.5	0.019^c^
Operation site	Left	139 (51.5)	19 (46.3)	0.616
	Right	131 (48.5)	22 (53.7)	
Reconstruction types	T/E	190 (70.4)	21 (51.2)	< 0.001
	DTI	5 (1.9)	20 (48.8)	
	TRAM	73 (27.0)	0 (0.0)	
	LD	2 (0.7)	0 (0.0)	
Incision types	IMF	51 (18.9)	0 (0.0)	< 0.001
	Radial	32 (11.9)	0 (0.0)	
	Upper-periareolar with extension	120 (44.4)	0 (0.0)	
	Lower-periareolar with extension	52 (19.3)	0 (0.0)	
	Circumareolar	3 (1.1)	0 (0.0)	
	Elliptical	12 (4.4)	0 (0.0)	
	Lateral or axillary	0 (0.0)	41 (100.0)	
SLNB^a^	No	20 (7.7)	2 (5.9)	>0.99
	Yes	239 (92.3)	32 (94.1)	
ALND^a^	No	224 (86.5)	31 (91.2)	0.592
	Yes	35 (13.5)	3 (8.8)	
Margin status^a^	No	240 (96.8)	33 (97.1)	0.423
	Yes	3 (1.2)	1 (2.9)	

Values are represented as mean ± SD or number (percentage).

ALND, axillary lymph node dissection; CNSM, conventional nipple-sparing mastectomy; DTI, direct-to-implant; IMF, inframammary fold; LD, latissimus dorsi flap; RNSM, robot-assisted nipple-sparing mastectomy; SLNB, sentinel lymph node biopsy; T/E, tissue expander; TRAM, transverse rectus abdominis musculocutaneous flap.

^a^29 cases of benign disease or BRCA mutation carriers were not included (n = 282).

^b^Chi-square test or Fisher’s exact test.

**^c^** Student’s t test or Mann–Whitney test.

T/E was the most common method for immediate reconstruction in both groups (**Table 2**). TRAM is the second most common method for immediate reconstruction in the CNSM group. Approximately half of the patients underwent DTI after RNSM.

Incision types are described in **Table 2**. Periareolar with extension was the most common incision in the CNSM group, followed by IMF, radial, elliptical, and curvilinear incision. Lateral or axillary incision was only used in the RNSM group. Incision types between the two groups were significantly different (*p* < 0.001). There was no significant difference of margin status between two groups. The CNSM group included one nipple and two superficial margins of tumor involvement. The RNSM group had one superficial margin involvement of tumor (**Table 2**). One patient who underwent RNSM showed false negative in subareolar mass in frozen section. Because the final pathology revealed invasive ductal carcinoma in the mass, NAC was sacrificed.

**Figure 3:**
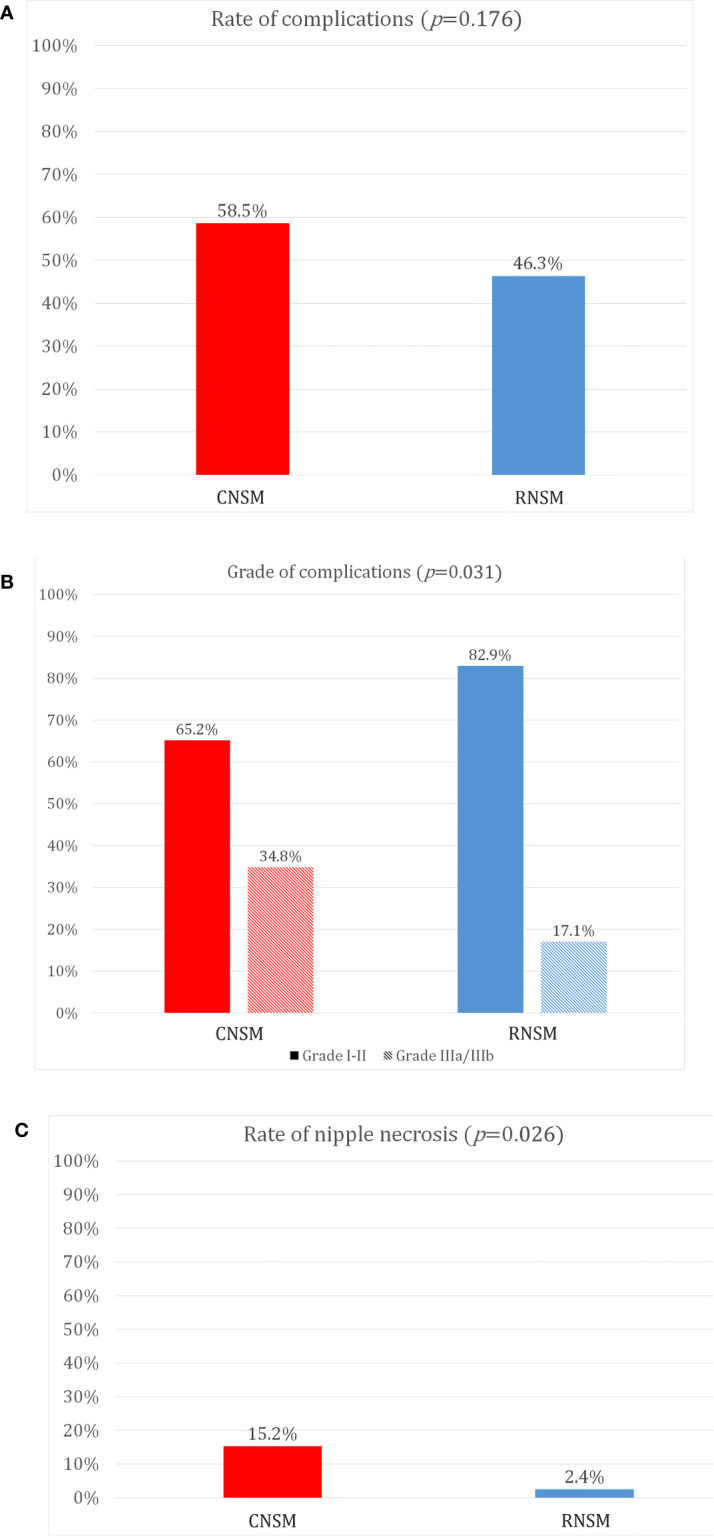
Comparison of post-operative complications between conventional nipple-sparing mastectomy and robot-assisted nipple sparing mastectomy. **(A)** Rate of complications, **(B)** Grade of complications, **(C)** Rate of nipple necrosis.

**Figure 3** shows grades of post-operative complications and nipple necrosis rates between the two groups. Post-operative complication rates were not different between the CNSM and RNSM groups (*p* = 0.176, 58.5 *vs.* 46.3%). There was no significant difference in implant loss and infection rates between the groups (for implant loss, *p* = 0.347, 0.7% for the CNSM group *vs.* 2.4% for the RNSM group, for infection, *p* = 0.101, 2.2% for the CNSM group *vs.* 7.3% for the RNSM group, data not shown). Post-operative complications requiring surgical intervention, such as wound revision, drain re-insertion, fat graft injection for volume defects, and implant removal were more common in the CNSM group (*p* = 0.031, grade ≥III, 34.8% *vs.* 17.1%). Nipple necrosis rate was significantly lower in the RNSM group than in the CNSM group (*p* = 0.026, 2.4 *vs.* 15.2%).

Multivariate analysis was conducted to evaluate risk factors related to high-grade complications. The rate of high-grade complications (grade ≥III) was statistically associated with the methods of the mastectomy and the operation time (*p* = 0.046 and *p* < 0.001) (**Table 3**).

**Table 3:** Multivariate analysis for risk factors related with high-grade complications.

		Clavien-Dindo Classification ≥Grade III	
		OR (95% CI)	*p*-value
Age (≤50 *vs.* >50)		0.751 (0.381–1.480)	0.408
Breast volume (≤310 g *vs.* >310 g)		1.638 (0.862–3.111)	0.132
Ptosis (Normal *vs.* Ptotic)		0.904 (0.489–1.673)	0.748
Operation time (min)		1.005 (1.004–1.007)	<0.001
Operation method (CNSM *vs.* RNSM)		0.406 (0.167–0.986)	0.046

CNSM, conventional nipple-sparing mastectomy; RNSM, robot-assisted nipple-sparing mastectomy.

## Discussion

Our study demonstrated the advantage of RNSM compared to the CNSM in terms of nipple necrosis rate. Previous studies suggested that certain incision types are significantly associated with nipple necrosis because the viability of the NAC is mainly maintained by blood supply from dermal layers (4, 30). Another study presented that a transaxillary incision could be the incision of choice for NSM with valid, oncological safe, and excellent cosmetic results in breast cancer patients or *BRCA* mutation carriers (31). For this reason, small axillary or lateral incisions in RNSM may have beneficial effects on the integrity of overlying skin and the NAC.

The rate of complications was not statistically different between the RNSM and the CNSM groups. Grades of post-operative complications were significantly different between the two groups. Compared to CNSM, RNSM showed lower rates of high-grade complications in the univariate and multivariate analysis. This different rate of high-grade complication may be due to different types of immediate reconstruction procedures. A previous study in our institution reported that reconstruction with TRAM free flap, LD flap with implant, and DTI presented with more post-operative NAC necrosis than reconstruction with a T/E (1). Similarly, another study reported that higher grades of post-operative complications occurred more commonly in patients with autologous reconstructions compared to those with implant-based reconstructions (32). In the present study, approximately one third (27.7%) of patients in the CNSM group underwent autologous reconstructions, and this may influence the higher grade of post-operative complications in this group. Therefore, it is important to consider types of reconstruction procedure as a stratification factor when conducting randomized clinical trials in the future.

In the present study, RNSM was mainly performed on patients with small- to medium-sized breasts without ptosis. This is concordant with previous studies (19). Toesca et al. mainly enrolled women with small- to medium-sized breasts with low grade ptosis in their randomized clinical trial (19, 33). This may be due to the fact that implant-based reconstruction is suitable for small- to medium-sized breasts with low grade ptosis. Implant-based reconstructions constituted the major reconstruction method after RNSM because LD or TRAM flap requires additional incisions compared to implant-based reconstruction. Autologous reconstruction after RNSM remained as a technical challenge of robotic surgery.

**Figure 4:**
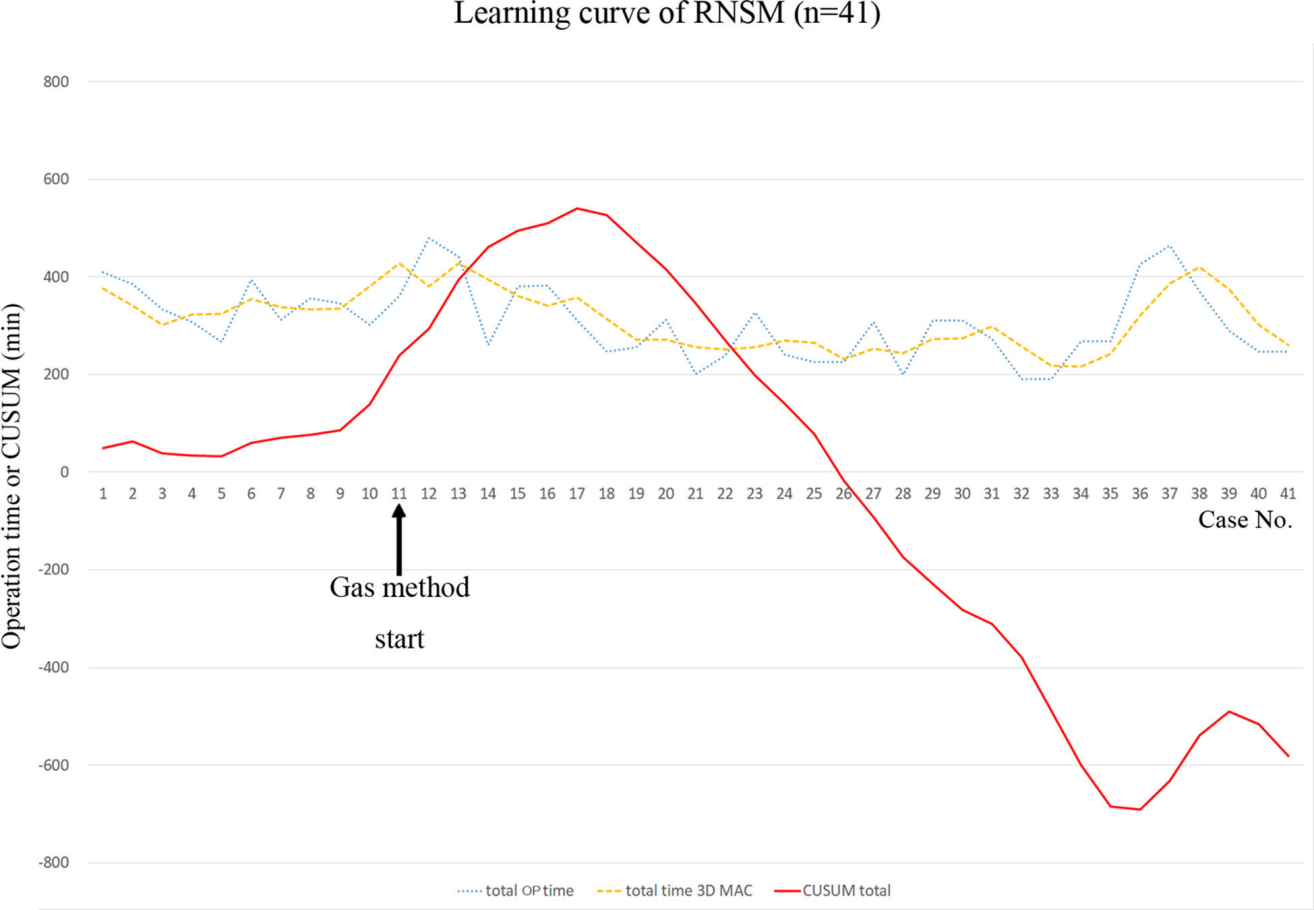
Learning curve of robot-assisted nipple-sparing mastectomy. *CUSUM*, cumulative sum technique; *OP*, operation; *RNSM*, robot-assisted nipple-sparing mastectomy; *3D MAC*, 3-day moving average curve.

Operation times for mastectomy were longer in the RNSM group than in the CNSM group in this study. Robotic surgery, including thyroidectomy, colectomy, and gastrectomy, presented with longer operation times than conventional surgery (34–36). This is due to the development of the working space, the robot docking, and surgeon’s experience (37). This is also the case with RNSM. Mean mastectomy time in the RNSM group was 181.5 min, and it was longer than mastectomy time in the CNSM group (95.5 min). However, as RNSM is a new technique, there was a learning period in the initial cases in this study. Even though operation times during RNSM decreased over time (**Figure 4**), a significant learning curve associated with a new technique such as RNSM may account for longer operation times compared to conventional procedures. Despite increased duration of mastectomy, console time in RNSM was approximately 1 hour (**Table 2**). Further studies regarding learning curves are necessary for comparisons of the two groups in terms of duration of operation.

Hospital stays were longer in the RNSM group than in the CNSM group. However, with a difference of only two days, there was no significant impact on clinical outcomes because there are differences in hospital stays according to surgeons’ preferences (data not shown).

There are several limitations to this study. The retrospective design of this study may have led to selection bias. Propensity matching would be an alternative method to reduce the limitations of a retrospective study. Also, the numeric disparity between the RNSM and CNSM groups was another limitation. Patient satisfaction and cosmetic outcomes, which may be one of the main advantages of RNSM, were not measured. A lack of detailed information on reconstructive techniques, such as subpectoral or prepectoral techniques, was another limitation of the study. Oncologic outcomes, such as loco-regional recurrence-free survival, disease-free survival, and overall survival, are important end-points in the treatment of patients with breast cancer. Prospective studies with longer follow-ups are needed to overcome these limitations. However, to the best of our knowledge, this is the largest study to evaluate differences in terms of grades of complications and rates of nipple necrosis between RNSM and CNSM. Moreover, the results of the current study support the feasibility and safety of robotic mastectomy as a treatment option for women with breast cancer or *BRCA* mutations.

## Conclusion

This study indicated that RNSM may have some advantages in terms of lower nipple necrosis and grade of post-operative complications. Further multicenter studies evaluating the clinical implications of RNSM should be conducted in the future.

## Data Availability Statement

The original contributions presented in the study are included in the article/supplementary material. Further inquiries can be directed to the corresponding author.

## Ethics Statement

The studies involving human participants were reviewed and approved by the institutional review board at Severance Hospital (4–2019–0510). Written informed consent for participation was not required for this study in accordance with the national legislation and the institutional requirements.

## Author Contributions

HSP, a principal investigator, conceived the ideas of the study. JL and HSP wrote the manuscript, and conducted the data analysis and interpretation. All authors contributed to the article and approved the submitted version.

## Conflict of Interest

HP received honoraria including consulting fee and travel support from Intuitive Surgical Korea and a research grant from Intuitive Surgical, Inc. which were not related with the data and analysis of the current study.

The remaining authors declare that the research was conducted in the absence of any commercial or financial relationships that could be construed as a potential conflict of interest.
