# Corrigendum: Post-operative complications and nipple necrosis rates between conventional and robotic nipple-sparing mastectomy

**DOI:** 10.3389/fonc.2022.985507

**Published:** 2022-08-16

**Authors:** Jeea Lee, Hyung Seok Park, Haemin Lee, Dong Won Lee, Seung Yong Song, Dae Hyun Lew, Jee Ye Kim, Seho Park, Seung Il Kim

**Affiliations:** ^1^ Department of Surgery, Yonsei University College of Medicine, Seoul, South Korea; ^2^ Department of Plastic and Reconstructive Surgery, Yonsei University College of Medicine, Seoul, South Korea

**Keywords:** breast neoplasms, robotic mastectomy, nipple-sparing mastectomy, minimal invasive surgery, nipple necrosis

In the published article, there was an error in **Table 2** as published. The margin status of pathology in the table was 240 (96.8%) in the CNSM group and 32 (94.1%) in the RNSM group (*p* = 0.404). The correct values of margin status are 245 (98.8%) in the CNSM group and 33 (97.1%) in the RNSM group (*p* = 0.423). The corrected **Table 2** Surgical methods and post-operative outcomes and its caption “Surgical methods and post-operative outcomes” appear below.

**Table 2:** Surgical methods and post-operative outcomes.

		CNSM	RNSM	*p*-value^b^
		(n = 270)	(n = 41)	
Hospital stay (days)		12 ± 3	14 ± 4	0.001^c^
Total operation time (min)		303.9 ± 195.9	308.9 ± 75.5	< 0.001 ^c^
Mastectomy time (min)		104.5 ± 40.5	181.5 ± 44.7	< 0.001 ^c^
Console time (min)		–	64 ± 40	–
Reconstruction time (min)		196.8 ± 182.5	140.5 ± 52.5	0.019 ^c^
Operation site	Left	139 (51.5)	19 (46.3)	0.616
	Right	131 (48.5)	22 (53.7)	
Reconstruction types	T/E	190 (70.4)	21 (51.2)	< 0.001
	DTI	5 (1.9)	20 (48.8)	
	TRAM	73 (27.0)	0 (0.0)	
	LD	2 (0.7)	0 (0.0)	
Incision types	IMF	51 (18.9)	0 (0.0)	< 0.001
	Radial	32 (11.9)	0 (0.0)	
	Upper-periareolar with extension	120 (44.4)	0 (0.0)	
	Lower-periareolar with extension	52 (19.3)	0 (0.0)	
	Circumareolar	3 (1.1)	0 (0.0)	
	Elliptical	12 (4.4)	0 (0.0)	
	Lateral or axillary	0 (0.0)	41 (100.0)	
SLNB^a^	No	20 (7.7)	2 (5.9)	> 0.99
	Yes	239 (92.3)	32 (94.1)	
ALND^a^	No	224 (86.5)	31 (91.2)	0.592
	Yes	35 (13.5)	3 (8.8)	
Margin status^a^	No	245 (98.8)	33 (97.1)	0.423
	Yes	3 (1.2)	1 (2.9)	

Values are represented as mean ± SD or number (percentage). ALND, axillary lymph node dissection; CNSM, conventional nipple-sparing mastectomy; DTI, direct-to-implant; IMF, inframammary fold; LD, latissimus dorsi flap; RNSM, robot-assisted nipple-sparing mastectomy; SLNB, sentinel lymph node biopsy; T/E, tissue expander; TRAM, transverse rectus abdominis musculocutaneous flap.

a 29 cases of benign disease or BRCA mutation carriers were not included (n = 282).

b Chi-square test or Fisher’s exact test.

c Student’s t test or Mann Whitney test.

The authors apologize for this error and state that this does not change the scientific conclusions of the article in any way. The original article has been updated.

## Publisher’s note

All claims expressed in this article are solely those of the authors and do not necessarily represent those of their affiliated organizations, or those of the publisher, the editors and the reviewers. Any product that may be evaluated in this article, or claim that may be made by its manufacturer, is not guaranteed or endorsed by the publisher.

